# Nutrition-related diseases and cardiovascular mortality in American society: national health and nutrition examination study, 1999–2006

**DOI:** 10.1186/s12889-022-14257-8

**Published:** 2022-10-03

**Authors:** Weihua Chen, Shanshan Shi, Jiabin Tu, Lihua Liao, Ying Liao, Kaihong Chen, Liling Chen, Rongchong Huang

**Affiliations:** 1 Department of Cardiology, Longyan First Affiliated Hospital of Fujian Medical University, Longyan, 364000 China; 2 Department of Cardiology, Beijing Friendship Hospital, Capital Medical University, Beijing, 100053 China

**Keywords:** Cardiovascular mortality, Malnutrition-sarcopenia syndrome, Nutrition-related diseases, National Health and Nutrition Examination Survey, Society

## Abstract

**Background:**

Despite many significant advances in treatment and management, cardiovascular disease remains the main cause of the global disease burden. Nutrition-related disease is a modifiable cardiovascular risk factor. However, few studies have examined the relationship between nutrition-related diseases and cardiovascular mortality.

**Objective:**

We aimed to investigate the association of nutrition-related diseases with cardiovascular mortality based on a large nationally representative community population.

**Design:**

We analyzed data from the National Health and Nutrition Examination Survey (NHANES) 1999–2006 with mortality follow-up through December 31, 2015. Finally, 12,469 participants were analyzed. Each participant was assigned to one of four groups: normal nutrition without sarcopenia, sarcopenia with normal nutrition, malnutrition without sarcopenia, and malnutrition-sarcopenia syndrome. Survival curves and Cox regressions based on the NHANES recommended weights were used to assess the association between nutrition-related diseases and cardiovascular mortality.

**Results:**

Of the 12,469 patients included in the study and divided into four groups, malnutrition-sarcopenia syndrome had the highest 5- and 10-year cardiovascular mortality rates. After adjustment for related factors, sarcopenia with normal nutrition (hazard ratio [HR]: 1.62, 95% confidence interval [CI]: 1.28–2.06; *P* < 0.001), malnutrition without sarcopenia (HR: 1.28, 95% CI:1.03–1.58; *P* = 0.024), and malnutrition-sarcopenia syndrome (HR: 2.66, 95% CI:1.89 − 3.74; *P* < 0.001) were significantly associated with increased risk of all-cause mortality. Malnutrition-sarcopenia syndrome remained associated with an increased risk of cardiovascular mortality (HR: 3.56, 95% CI: 1.17 − 10.84; *P* < 0.001).

**Conclusions:**

Malnutrition-sarcopenia syndrome was highly prevalent among community-dwelling adults in the United States and was a strong prognostic factor for cardiovascular mortality in the community setting. Randomized clinical trials are needed to demonstrate whether prevention or treatment of malnutrition-sarcopenia syndrome in community populations can reduce global cardiovascular mortality.

**Supplementary Information:**

The online version contains supplementary material available at 10.1186/s12889-022-14257-8.

## Introduction

Cardiovascular disease (CVD) is the leading cause of morbidity and mortality worldwide, accounting for 27% of global deaths [1, 2]. Despite many significant advances in treatment and management, CVD remains the main cause of the global disease burden [3].

There is a growing focus on primary prevention and on reducing the burden of CVD by treating other comorbidities, rather than focusing on the prevention and treatment of CVD itself. Lifestyle changes related to smoking, diet, and exercise can prevent premature cardiovascular events [4, 5]. Recent studies have shown that treating conditions such as hyperlipidemia or depression could reduce cardiovascular mortality [6–8]. Therefore, it is possible to prevent, detect, and treat other diseases associated with cardiovascular mortality to reduce the global burden of CVD.

Malnutrition and sarcopenia are closely related to premature death and cardiovascular mortality [9, 10]. Similarly, recent studies have shown that malnutrition-sarcopenia syndrome is also associated with high all-cause mortality [11, 12]. The advantage of nutrition-related disease over other clinical variables is that it is a modifiable risk factor that physicians can act on [13]. However, few studies have examined the relationship between nutrition-related diseases and cardiovascular mortality.

This study aimed to investigate the association of nutrition-related diseases with cardiovascular mortality based on a large nationally representative community population and further establish a theoretical basis for the possibility to prevent or reduce global cardiovascular mortality.

## Methods

### Study population

**Fig. 1:**
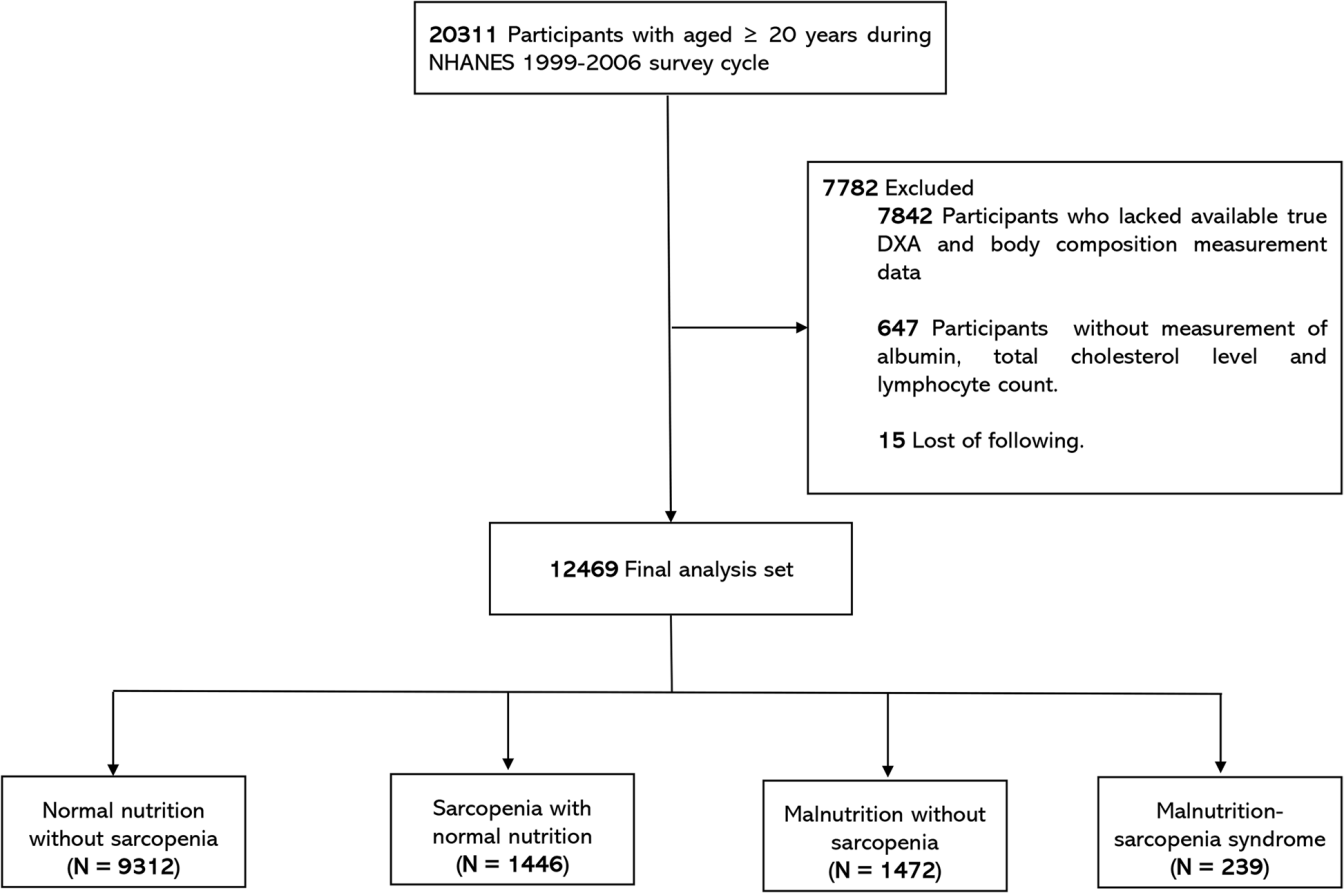
Flowchart of study design

National Health and Nutrition Examination Survey (NHANES) is a nationally representative health survey designed and administered by the National Center for Health Statistics at the Centers for Disease Control and Prevention. The NHANES was designed to represent the civilian non-institutionalized United States population using a complex multistage probability sampling methodology. We conducted a retrospective analysis of a cohort of the United States cohort of NHANES. As shown in Fig. 1, this study included participants ≥ 20 years of age during NHANES 1999 − 2006 (*n* = 20,311). Of these participants, 7,842 were excluded based on the lacked available true dual-energy x-ray absorptiometry (DXA) and body composition measurement data, lack of measured albumin, total cholesterol level and lymphocyte count, or loss to follow-up. Thus, 12,469 patients were enrolled in the present study.

### Screening for nutrition-related diseases

Whole body DXA scans were acquired using model QDR-4500A fan-beam densitometers (Hologic, Inc, Bedford, MA) in NHANES participants over 8 years of age. DXA exclusion criteria included pregnancy, weight > 300 pounds (136 kg, weight limit of the scanner), height over 6.5 feet (length of DXA table), use of the barium radiographic contrast material in the preceding 7 days, or nuclear medicine studies in the past 3 days.

Appendicular skeletal muscle mass was measured using DXA. The sarcopenia index was calculated as total appendicular skeletal muscle mass [(in kg)/body mass index (BMI, kg/m^2^)]. Sarcopenia was defined as the lowest quintile for sex-specific sarcopenia index cut-off values (0.789 for men and 0.512 for women), based on a modified recommendation from the Foundation for the National Institutes of Health [14].

The Controlling Nutritional Status (CONUT) score was the screening tool for the nutritional status [15]. CONUT takes into account serum albumin, cholesterol, and total lymphocyte count. Participants with a score of 0 to 1 were classified as normal nutrition status, while those with a score of 2 or more were considered malnutrition status. Moreover, we used the nutritional risk index (NRI) to define the nutrition status and performed a sensitivity analysis. Details of the two scoring systems are summarized in Supplementary Table 1.

### Definitions of variables of interest

Age, sex, race, education level, smoking status, alcohol use, and histories of congestive heart failure (CHF), coronary heart disease (CHD), diabetes mellitus (DM), hypertension, and cancer were self-reported. Diagnosis of comorbidities was based on an affirmative response to the question, “Has a doctor or other health professional ever told you that you had (CHF, CHD, DM, hypertension, and cancer)?”.

### Primary outcome

Mortality status was determined based on a probabilistic record match with the National Death Index using demographic identifiers. (Available at: http://www.cdc.gov/nchs/data/datalinkage/nh99+_mortality_matching_methodology_final.pdf. Accessed July 22, 2010.). The primary outcome was cardiovascular mortality. The secondary outcome was all-cause mortality. Cause of death was categorized using the International Classification of Diseases 10th edition (ICD-10). Cardiovascular mortality was categorized using ICD-10 codes I00–I078. For participants in NHANES 1999–2006, mortality follow-up data was available through December 31, 2015.

### Statistical analyses

NHANES recommended weights were used to account for planned oversampling of specific groups. The continuous variables are expressed as the mean ± standard deviation. Categorical variables are expressed as counts (percentages). Baseline characteristics between the four groups were compared using an ANOVA for continuous variables and a χ2 test for categorical variables.

Each patient was assigned to one of four groups: normal nutrition without sarcopenia, sarcopenia with normal nutrition, malnutrition without sarcopenia, and malnutrition-sarcopenia syndrome. To evaluate the association between nutrition-related diseases and mortality, we used Kaplan–Meier estimates based on the NHANES recommended weights to calculate cumulative survival probabilities for cardiovascular mortality and all-cause mortality, and univariate and multivariate cox regression analyses based on the NHANES recommended weights. Hazard ratio (HR) and 95% confidence interval (CI) were calculated. Model 1 was a crude model unadjusted for potential confounders. Model 2 was adjusted for demographic factors, including age, sex, and race/ethnicity. Model 3 was further adjusted for education level, smoking status, alcohol use, BMI, CHF, CHD, DM, hypertension, and cancer. To further investigate the reliability of our study, we defined nutrition status differently and conducted sensitivity analyses. We further explored the relationship between nutrition-related diseases and cardiovascular mortality in different subgroups (age, sex, and obesity).

All analyses were performed using R software (version 4.0.3; R Foundation for Statistical Computing, Vienna, Austria). A two-sided *P*-value < 0.05 indicated the significance for all analyses.

## Results

### Patient characteristics

In total, 12,469 participants remained in the study. They were divided into four groups based on the combined presence of malnutrition or sarcopenia. The distribution of the four groups were normal nutrition without sarcopenia (*n* = 9,312), sarcopenia with normal nutrition (*n* = 1,446), malnutrition without sarcopenia (*n* = 1,472), and malnutrition-sarcopenia syndrome (*n* = 239), respectively. Participants in the malnutrition-sarcopenia syndrome were older (weighted, 62.0 ± 1.2 years) and less likely to be female (weighted, 34.9%). Non-Hispanic Blacks were more prevalent in the malnutrition without sarcopenia group (weighted,11.9%) and less prevalent in the sarcopenia with normal nutrition group (weighted,1.8%). Concerning BMI, compared to normal nutrition without sarcopenia (weighted, 27.3 ± 0.1 kg/m^2^) and malnutrition without sarcopenia (weighted, 25.4 ± 0.2 kg/m^2^), BMI was significantly higher for those in the sarcopenia with normal nutrition group (weighted, 31.5 ± 0.2 kg/m^2^) and malnutrition-sarcopenia syndrome group (weighted, 31.3 ± 0.4 kg/m^2^). Participants in the malnutrition-sarcopenia syndrome group were more likely to have hypertension (weighted, 54.9%) and DM (weighted, 22.7%), CHD (weighted, 16.8%), CHF (weighted, 12.3%), and cancer (weighted, 17.8%). The baseline characteristics of the study subjects at entry are summarized in Table 1.

**Table 1:** Baseline characteristics of the study population (weighted)

	Normal nutrition without sarcopenia (*n* = 9312)	Sarcopenia with normal nutrition (*n* = 1446)	Malnutrition without sarcopenia (*n* = 1472)	Malnutrition-sarcopenia syndrome (*n* = 239)	*P*-value
Age	44.1 ± 0.2	56.5 ± 0.5	42.3 ± 0.5	62.0 ± 1.2	< 0.001
Female	4694 (51.4)	681 (48.3)	621 (47.3)	88 (34.9)	< 0.001
Race					
Mexican American	1902 (7.1)	664 (16.6)	250 (6.4)	89 (12.1)	< 0.001
Other Hispanic	421 (5.3)	89 (9.7)	43 (3.5)	8 (4.1)	
Non-Hispanic White	4801 (73.6)	603 (65.7)	771 (73.4)	124 (75.4)	
Non-Hispanic Black	1858 (9.8)	45 (1.8)	349 (11.9)	8 (2.2)	
Other Race	330 (4.5)	45 (6.3)	59 (4.9)	10 (6.3)	
Education levels					
< 12	4780 (42.9)	1057 (62.7)	741 (41.0)	157 (55.7)	< 0.001
12	2607 (30.9)	253 (24.4)	410 (31.0)	51 (30.3)	
> 12	1915 (26.1)	132 (12.9)	319 (28.0)	31 (14.0)	
Smoking status	4642 (51.1)	717 (50.1)	664 (42.9)	134 (55.8)	< 0.001
Alcohol use	4593 (32.5)	697 (40.0)	738 (33.8)	160 (50.6)	0.003
BMI	27.3 ± 0.1	31.5 ± 0.2	25.4 ± 0.2	31.3 ± 0.4	< 0.001
Hypertension	2500 (23.4)	603 (42.6)	406 (21.1)	126 (54.9)	< 0.001
DM	674 (4.9)	254 (15.8)	137 (5.6)	55 (22.7)	< 0.001
CHD	253 (1.9)	108 (8.2)	92 (4.7)	30 (16.8)	< 0.001
CHF	155 (1.1)	78 (5.3)	54 (2.4)	28 (12.3)	< 0.001
Cancer	589 (6.2)	126 (8.9)	162 (8.8)	41 (17.8)	< 0.001

*Abbreviations*: *BMI* Body mass index, *DM* Diabetes mellitus, *CHD* Coronary heart disease, *CHF* Congestive heart failure

### All-cause and cardiovascular mortality

**Fig. 2:**
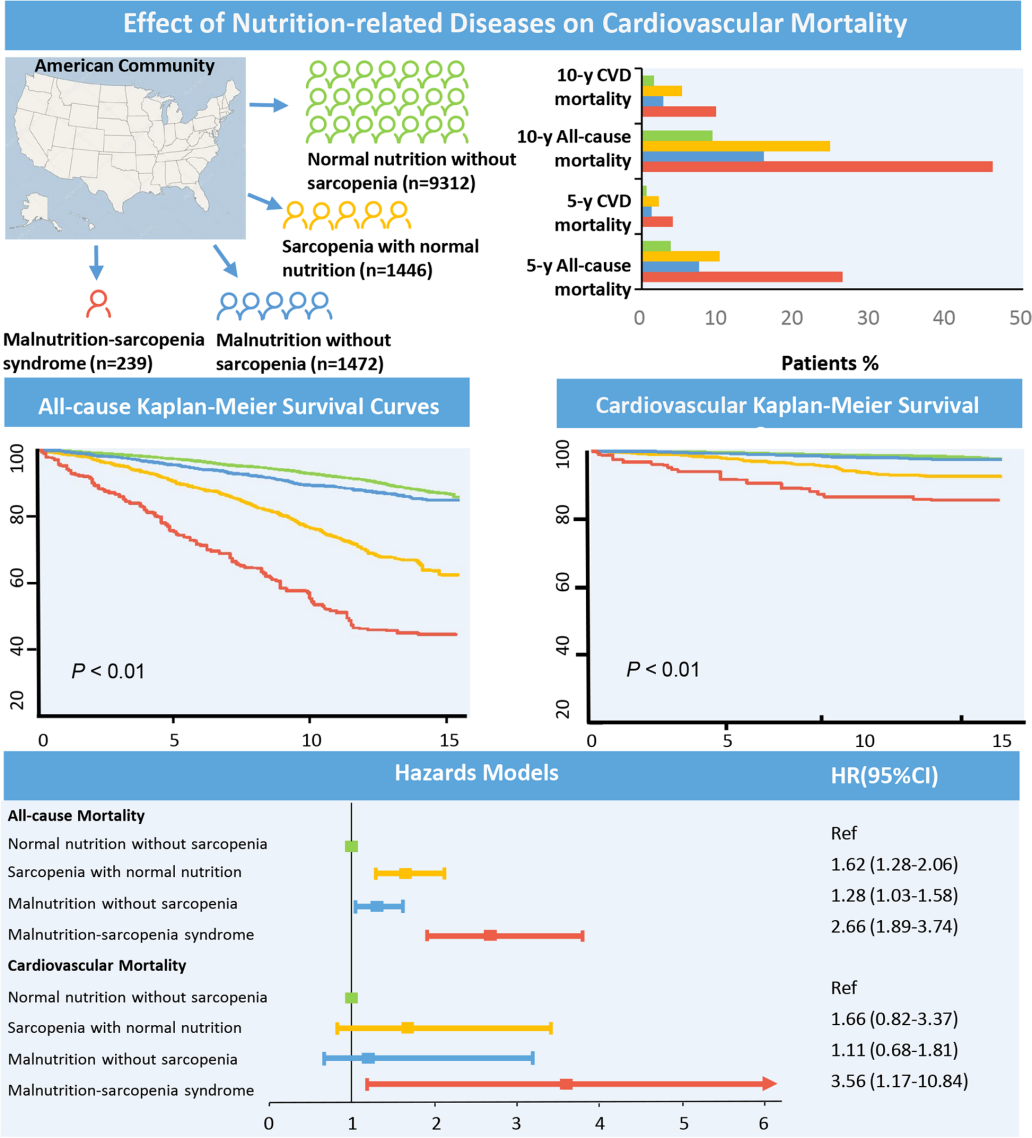
Effect of nutrition-related diseases on cardiovascular mortality

Malnutrition-sarcopenia syndrome had the highest 5- and 10-year cardiovascular mortality rates (Fig. 2). Kaplan–Meier survival analysis curves revealed significantly lower long-term survival rates for patients with any form of malnutrition and sarcopenia, including malnutrition-sarcopenia syndrome, compared with patients with no malnutrition and sarcopenia (*P* < 0.01, Fig. 2). Univariate Cox proportional risk analysis revealed independent associations with increased risk of all-cause mortality for sarcopenia with normal nutrition (weighted, HR: 3.52, 95% CI: 3.05–4.06; *P* < 0.001), malnutrition without sarcopenia (weighted, HR: 1.34, 95% CI: 1.14–2.97; *P* < 0.001), and malnutrition-sarcopenia syndrome (weighted, HR: 7.81, 95% CI: 6.07–10.03; *P* < 0.001). Independent association with increased risk of cardiovascular mortality was evident for sarcopenia with normal nutrition (weighted, HR: 4.66, 95% CI: 3.20–6.78; *P* < 0.001) and malnutrition-sarcopenia syndrome (weighted, HR: 10.67, 95% CI: 5.60–20.32; *P* < 0.001). Even after adjusting for age, gender, race, education level, smoking status, alcohol use, BMI, CHF, CHD, DM, hypertension, and cancer, significant associations with increased risk of all-cause mortality were evident for sarcopenia with normal nutrition (weighted, HR: 1.62, 95% CI:1.28 − 2.06; *P* < 0.001), malnutrition without sarcopenia (weighted, HR: 1.28, 95% CI: 1.03–1.58; *P* = 0.024) and malnutrition-sarcopenia syndrome (weighted, HR: 2.66, 95% CI: 1.89–3.74; *P* < 0.001). Malnutrition-sarcopenia syndrome remained associated with an increased risk of cardiovascular mortality (weighted, HR: 3.56, 95% CI: 1.17–10.84; *P* = 0.025; Table 2). Sensitivity analysis that used the NRI score also showed similar results (Supplementary Table 2).

**Table 2:** All-cause and cardiovascular mortality hazard ratios (HRs) for participants aged 20 years and older according to nutrition-related diseases: NHANES survey 1999 to 2006 with follow-up through 2015^a^

Models	All-cause mortality			Cardiovascular mortality		
	HR	95% CI	*P*-value	HR	95% CI	*P*-value
Model 1						
(Normal nutrition without sarcopenia as reference)						
Sarcopenia with normal nutrition	3.52	3.05–4.06	< 0.001	4.66	3.20–6.78	< 0.001
Malnutrition without sarcopenia	1.34	1.14–2.97	< 0.001	1.43	0.97–2.11	0.070
Malnutrition-sarcopenia syndrome	7.81	6.07–10.03	< 0.001	10.67	5.60–20.32	< 0.001
Model 2						
(Normal nutrition without sarcopenia as reference)						
Sarcopenia with normal nutrition	1.40	1.22–1.61	< 0.001	1.44	0.97–2.16	0.074
Malnutrition without sarcopenia	1.30	1.11–1.53	0.001	1.29	0.93–1.80	0.131
Malnutrition-sarcopenia syndrome	2.31	1.82–2.93	< 0.001	2.41	1.18–4.91	0.015
Model 3						
(Normal nutrition without sarcopenia as reference)						
Sarcopenia with normal nutrition	1.62	1.28–2.06	< 0.001	1.66	0.82–3.37	0.163
Malnutrition without sarcopenia	1.28	1.03–1.58	0.024	1.11	0.68–1.81	0.672
Malnutrition-sarcopenia syndrome	2.66	1.89–3.74	< 0.001	3.56	1.17–10.84	0.025

Model 1: No adjusted

Model 2: Adjusted by age, gender, race/ethnicity

Model 3: Adjusted by age, gender, race/ethnicity, education level, smoking status, alcohol use, BMI, CHF, CHD, DM, hypertension, Cancer

^a^Nutrition status was defined by the controlling nutritional status score

### Subgroups

When participants were younger than 65 years, malnutrition-sarcopenia syndrome remained associated with an increased risk of cardiovascular mortality (weighted, adjusted HR: 7.10, 95% CI: 1.38–36.64; *P* = 0.019). Furthermore, a significant association was evident between malnutrition-sarcopenia syndrome and cardiovascular mortality in non-obese participants (weighted, adjusted HR: 4.03, 95% CI: 1.44–11.29; *P* = 0.008; Table 3).

**Table 3:** All-cause and cardiovascular mortality hazard ratios (HRs) in different subgroups^a^

Variable	All-cause mortality			Cardiovascular mortality		
	HR	95% CI	*P*-value	HR	95% CI	*P*-value
Age						
< 65						
(Normal nutrition without sarcopenia as reference)						
Sarcopenia with normal nutrition	2.61	1.81–3.77	< 0.001	1.26	0.41–3.90	0.681
Malnutrition without sarcopenia	1.35	0.94–1.94	0.100	1.13	0.42–3.02	0.810
Malnutrition-sarcopenia syndrome	5.77	3.21–10.37	< 0.001	7.10	1.38–36.64	0.019
≥ 65						
(Normal nutrition without sarcopenia as reference)						
Sarcopenia with normal nutrition	1.86	1.39–2.49	< 0.001	1.94	0.88–4.27	0.098
Malnutrition without sarcopenia	1.12	0.87–1.44	0.389	1.17	0.63–2.17	0.619
Malnutrition-sarcopenia syndrome	2.97	1.81–4.88	< 0.001	2.56	0.90–7.31	0.078
Sex						
Male						
(Normal nutrition without sarcopenia as reference)						
Sarcopenia with normal nutrition	1.65	1.24–2.19	< 0.001	1.48	0.62–3.53	0.380
Malnutrition without sarcopenia	1.25	0.98–1.58	0.072	1.14	0.65–2.00	0.655
Malnutrition-sarcopenia syndrome	2.78	1.92–4.02	< 0.001	3.35	0.87–12.95	0.080
Female						
(Normal nutrition without sarcopenia as reference)						
Sarcopenia with normal nutrition	1.58	1.06–2.36	0.025	2.50	1.17–5.34	0.018
Malnutrition without sarcopenia	1.53	1.05–2.24	0.028	1.61	0.70–3.71	0.262
Malnutrition-sarcopenia syndrome	2.55	1.11–5.85	0.027	4.53	0.51–40.21	0.175
Obesity						
Non-obese						
(Normal nutrition without sarcopenia as reference)						
Sarcopenia with normal nutrition	1.80	1.38–2.33	< 0.001	1.64	0.70–3.86	0.254
Malnutrition without sarcopenia	1.35	1.06–1.72	0.017	1.21	0.65–2.25	0.549
Malnutrition-sarcopenia syndrome	3.50	2.61–4.70	< 0.001	4.03	1.44–11.29	0.008
Obese						
(Normal nutrition without sarcopenia as reference)						
Sarcopenia with normal nutrition	1.33	0.89–1.98	0.166	1.28	0.46–3.50	0.632
Malnutrition without sarcopenia	1.08	0.67–1.73	0.747	0.96	0.30–3.11	0.944
Malnutrition-sarcopenia syndrome	1.71	0.74–3.91	0.207	2.67	0.35–20.05	0.341

Analyses were adjusted for age, gender, race/ethnicity, education level, smoking status, alcohol use, BMI, CHF, CHD, DM, hypertension, Cancer

^a^Nutrition status was defined by the controlling nutritional status score

## Discussion

In this large retrospective study of community populations in the United States, malnutrition-sarcopenia syndrome was associated with an increased risk of cardiovascular mortality and all-cause mortality. Compared to patients without malnutrition and sarcopenia, patients with malnutrition-sarcopenia syndrome had an almost fourfold increased risk of cardiovascular mortality and an almost threefold increased risk of all-cause mortality. Even though both sarcopenia with normal nutrition and malnutrition without sarcopenia were associated with an increased risk of all-cause mortality, neither sarcopenia with normal nutrition alone nor malnutrition without sarcopenia increased the risk of cardiovascular mortality.

CVD is the leading cause of death worldwide [16]. To reduce the burden of CVD, many studies have proposed various interventions. Nutrition-related diseases are an important risk factor for death from CVD [17]. Furthermore, hospitalized patients with nutrition-related diseases have a higher risk of cardiovascular mortality than those without nutrition-related diseases [18, 19]. Malnutrition and sarcopenia are prevalent in community populations [20] and always occur together. Therefore, there is an urgent need to explore whether controlling nutrition-related diseases can reduce cardiovascular mortality to help reduce the global burden of CVD.

Our results suggested that malnutrition-sarcopenia syndrome was independently associated with an increased risk of all-cause and cardiovascular mortality in community populations in the United States. Sarcopenia is considered an important risk factor for cardiovascular mortality in community-dwelling women [21]. Consistent with the latter study, we observed that sarcopenia alone also affected cardiovascular mortality in females. Moreover, sarcopenia combined with malnutrition was an important risk factor for cardiovascular mortality in younger participants. The prevalence of obesity has increased dramatically over the past three decades [22]. Prior data have associated obesity and nutrition-related diseases in individuals with CVD [23]. Therefore, we performed an exploratory subgroup analysis of obesity and found that malnutrition-sarcopenia syndrome influenced cardiovascular mortality in the non-obese subgroup. The association between malnutrition-sarcopenia syndrome and cardiovascular mortality in obese participants did not seem to be statistically significant. However, we cannot exclude that the association does not apply to this group of participants. Furthermore, considering the high prevalence of nutrition-related diseases and obesity in the community population, this area deserves further investigation. Regardless, we suggest that the incidence of cardiovascular mortality can be reduced by screening and intervention for malnutrition-sarcopenia syndrome in community populations.

Malnutrition leads to CVD in a number of ways. For example, malnutrition can cause autonomic nervous imbalances, leading to high blood pressure and an increased heart rate [24]. Malnutrition can also cause insulin resistance and low-grade systemic inflammatory response, which can accelerate the progression of coronary atherosclerosis [25, 26]. In addition to malnutrition, sarcopenia can lead to CVD through these pathways. Furthermore, in addition to being associated with inflammation, sarcopenia can negatively affect the body by affecting the distribution of glucose in the muscles [27–29]. Previous studies have shown that poor nutrition can worsen sarcopenia, which in turn can worsen the body's nutritional status, forming a vicious cycle [30–32]. This cycle may further increase the risk of cardiovascular mortality in patients with malnutrition-sarcopenia syndrome.

Our study confirmed that malnutrition-sarcopenia syndrome was an independent factor of CVD adverse events in community populations. Therefore, we recommend screening for malnutrition-sarcopenia syndrome in community populations. Interestingly, patients with malnutrition-sarcopenia syndrome in nursing homes display higher all-cause mortality than the consecutively admitted older patients with malnutrition-sarcopenia syndrome [12, 33]. Perhaps, hospitals pay more attention to patients' nutritional status than nursing homes do. In support of this idea, we found that the consecutively admitted older patients are more likely to use nutritional supplements than patients from nursing homes. The benefits of nutritional supplements for malnutrition and sarcopenia have been previously demonstrated [34, 35]. In addition, appropriate nutritional interventions can prevent and reverse atherosclerosis, and lessen CVD risk factors [36]. Therefore, we recommend enhanced use of nutritional supplements for those who develop nutrition-related diseases in the community. Because community-dwelling individuals tend to be more athletic than hospitalized patients, we also recommend exercise interventions in the community, which can maintain or enhance muscle mass [37].

The strength of this study was its novelty in exploring the relationship between nutrition-related disease and cardiovascular mortality using a large national community sample, which makes the results generalizable. There were several limitations of this study. The primary limitation was that we did not collect handgrip strength on study participants and only used appendicular skeletal muscle mass to define sarcopenia. However, this definition is commonly recognized and used in studies [38]. A second limitation was that information on comorbid health conditions was self-reported. This likely underestimated the prevalence of comorbidities. Another limitation is that the competing risk of cardiovascular mortality was not accounted for in our models. But with NHANES and the weighting, it seems difficult to balance them all at the same time. Finally, we used only the nutritional status risk score tool for the diagnosis of malnutrition. Thus, the data may be biased. However, we used two scoring tools to verify the stability of our study and nutritional status risk score tools have higher objectivity and accuracy compared with other evaluation methods.

## Conclusion

In summary, sarcopenia or malnutrition alone was not associated with cardiovascular mortality. However, malnutrition-sarcopenia syndrome was highly prevalent among community-dwelling adults in the United States and was a strong prognostic factor for premature cardiovascular mortality among these adults. This suggested that malnutrition-sarcopenia syndrome in community populations could likely be prevented or treated with some easily achievable measures. This would reduce global cardiovascular mortality. However, randomized clinical trials are necessary to demonstrate efficacy and to clarify this point.

## Supplementary Information

**Additional file 1:Supplementary Table 1.** Procedures for the evaluation of each nutritional index.

**Additional file 2:Supplementary Table 2.** Sensitivity analyses for all-cause and cardiovascular mortality hazard ratios (HRs) for participants aged 20 years and older according to nutrition-related diseases: NHANES survey 1999–2006 with follow-up through 2015. *

## Data Availability

All data are available as publicly accessible datasets through NHANES. It is open and publicly accessible through the following link; https://wwwn.cdc.gov/nchs/nhanes/.

## Abbreviations

CVD: Cardiovascular disease
NHANES: National Health and Nutrition Examination Survey
DXA: Dual-energy x-ray absorptiometry
CONUT: Controlling nutritional status
NRI: Nutritional risk index
CHF: Congestive heart failure
CHD: Coronary heart disease
DM: Diabetes mellitus
ICD-10: Classification of Diseases 10^th^ edition
HR: Hazard ratio
BMI: Body mass index
