# Supplementary material for: Nutrition-related diseases and cardiovascular mortality in American society: national health and nutrition examination study, 1999–2006
Source: BMC Public Health. 2022 Oct 3;22:1849. doi: 10.1186/s12889-022-14257-8 (PMC9531382; doi:10.1186/s12889-022-14257-8)
Supplement: Supplementary file 1 — Additional file 1:Supplementary Table 1. Procedures for the evaluation of each nutritional index. [file 12889_2022_14257_MOESM1_ESM.docx]

**Supplementary table 1. Procedures for the evaluation of each nutritional index.**

| Nutritional Indices | | Risk of Malnutrition | | | |
| --- | --- | --- | --- | --- | --- |
|  |  | Absent | Mild | Moderate | Severe |
| CONUT, points | | 0-1 | 2-4 | 5-8 | 9-12 |
| Formula | Albumin, g/dl (score) | ≥3.5 (0) | 3.0-3.4 (2) | 2.5-2.9 (4) | <2.5 (6) |
|  | Total cholesterol, mmol/l (score) | ≥180 (0) | 140-199 (1) | 100-139 (2) | <100 (3) |
|  | Lymphocyte count, x10^9^/l (score) | ≥1.60 (0) | 1.20-1.59 (1) | 0.80 - 1.19 (2) | <0.80 (3) |
| NRI, points | | ≥100 | 97.50-99.99 | 83.50-97.49 | <83.50 |
| Formula 1.489 x serum albumin (g/l) + 41.7 x (weight in kilograms/ideal weight) | | | | | |

Note: Procedures for the evaluation of each nutritional index.
