# Supplementary material for: Nutrition-related diseases and cardiovascular mortality in American society: national health and nutrition examination study, 1999–2006
Source: BMC Public Health. 2022 Oct 3;22:1849. doi: 10.1186/s12889-022-14257-8 (PMC9531382; doi:10.1186/s12889-022-14257-8)
Supplement: Supplementary file 2 — Additional file 2:Supplementary Table 2. Sensitivity analyses for all-cause and cardiovascular mortality hazard ratios (HRs) for participants aged 20 years and older according to nutrition-related diseases: NHANES survey 1999–2006 with follow-up through 2015. * [file 12889_2022_14257_MOESM2_ESM.docx]

**Supplementary Table 2. Sensitivity analyses for all-cause and cardiovascular mortality hazard ratios (HRs) for participants aged 20 years and older according to nutrition-related diseases: NHANES survey 1999–2006 with follow-up through 2015. ***

| **Models** | **All-cause mortality** | | |  | **Cardiovascular mortality** | | |
| --- | --- | --- | --- | --- | --- | --- | --- |
|  | **HR** | **95% CI** | ***P*-value** |  | **HR** | **95% CI** | ***P*-value** |
| **Model 1** |  |  |  |  |  |  |  |
| (Normal nutrition without sarcopenia as reference) |  |  |  |  |  |  |  |
| Sarcopenia with normal nutrition | 3.85 | 3.29-4.50 | <0.001 |  | 4.76 | 3.10-7.31 | <0.001 |
| Malnutrition without sarcopenia | 2.19 | 1.84-2.60 | <0.001 |  | 2.57 | 1.76-3.74 | <0.001 |
| Malnutrition-sarcopenia syndrome | 6.62 | 5.27-8.32 | <0.001 |  | 11.32 | 6.34-20.19 | <0.001 |
| **Model 2** |  |  |  |  |  |  |  |
| (Normal nutrition without sarcopenia as reference) |  |  |  |  |  |  |  |
| Sarcopenia with normal nutrition | 1.47 | 1.28-1.70 | <0.001 |  | 1.44 | 0.90-2.29 | 0.127 |
| Malnutrition without sarcopenia | 1.72 | 1.45-2.03 | <0.001 |  | 2.06 | 1.35-3.10 | <0.001 |
| Malnutrition-sarcopenia syndrome | 2.15 | 1.75-2.65 | <0.001 |  | 2.93 | 1.69-5.07 | <0.001 |
| **Model 3** |  |  |  |  |  |  |  |
| (Normal nutrition without sarcopenia as reference) |  |  |  |  |  |  |  |
| Sarcopenia with normal nutrition | 1.66 | 1.34-2.06 | <0.001 |  | 1.44 | 0.65-3.20 | 0.367 |
| Malnutrition without sarcopenia | 1.51 | 1.23-1.87 | <0.001 |  | 1.90 | 1.07-3.36 | 0.027 |
| Malnutrition-sarcopenia syndrome | 2.31 | 1.58-3.38 | <0.001 |  | 5.40 | 2.09-13.94 | <0.001 |

*: Nutrition status was defined by The Nutritional Risk Index.

Model 1: No adjustments.

Model 2: Adjusted by age, sex, and race.

Model 3: Adjusted by age, sex, race, education level, smoking status, alcohol use, BMI, CHF, CHD, DM, hypertension, and cancer.
